# Hemorrhagic Pleural Effusion: A Rare Presentation of Vitamin K Deficiency in an Adult Patient

**DOI:** 10.7759/cureus.11374

**Published:** 2020-11-07

**Authors:** Somto T Nwaedozie, Javad Najjar Mojarrab, Dillon C Svoboda, Abuzaid Medani

**Affiliations:** 1 Internal Medicine, Marshfield Clinic Health System, Marshfield, USA; 2 Internal Medicine, University of Wisconsin School of Medicine and Public Health, Madison, USA; 3 Hospital Medicine, Marshfield Medical Center, Marshfield, USA

**Keywords:** vitamin k deficiency, hemorrhagic pleural effusion, coagulopathy, depression, adult

## Abstract

Nutritionally acquired vitamin K deficiency is a rare condition in adults and can uncommonly present as hemorrhagic pleural effusion. We discuss the case of A 44-year-old apparently healthy man who presented with left-sided pleuritic chest pain two months after experiencing upper respiratory tract symptoms. Imaging revealed a loculated left-sided effusion, and a corresponding thoracentesis yielded exudative hemorrhagic fluid with no microbial growth. Laboratory work-up showed prolonged clotting time with low factors II, VII, and X activity, absence of clotting factor inhibitors, and very low serum vitamin K levels. A five-day course of oral vitamin K and nutritional optimization normalized the clotting profile.

Acquired vitamin K deficiency from poor micronutrient intake is rare in adults and can result in hemorrhagic pleural effusion. Vitamin K supplementation can normalize the clotting profile while nutritional counseling helps prevent a recurrence. Malnutrition-induced vitamin K deficiency can occur in the setting of a major depressive disorder in adults. Thorough patient history and physical examination are necessary to promptly identify and reverse the coagulopathy.

## Introduction

Vitamin K is a vital fat-soluble vitamin that plays a major role in the coagulation pathways by acting as an endogenous cofactor for the carboxylation and activation of certain coagulation factors as well as endogenous anticoagulants. Although vitamin K deficiency is common in neonates, it is rare in otherwise healthy adults due to the wide distribution of phylloquinone (vitamin K1) in plants, menaquinone (vitamin K2) production by gut microflora, and endogenous intracellular vitamin K recycling [1]. However, acquired vitamin K deficiency can occur in those with predisposing factors like prolonged fasting or starvation, hepatobiliary or pancreatic disease, intestinal mucosal dysfunction, use of medications like anticoagulants, broad-spectrum antibiotics, and high-dose vitamin A and E supplementation [1,2-7]. Although vitamin K deficiency can manifest clinically as bruising easily, mucosal bleeding, gastrointestinal bleeding, or hematuria, reports of vitamin K deficiency leading to hemorrhagic pleural effusion in an otherwise healthy adult are very rare [1,2]. In this report, we present a case of significant hemorrhagic pleural effusion in an adult with vitamin K deficiency.

## Case presentation

A 44-year-old male with a past medical history of hypertension, obesity, and depression presented to our facility on account of a left-sided chest and abdominal pain that had started five days before his index visit. The patient reported developing a gradual-onset, dull, moderately intense, and intermittent chest pain that radiated to his left abdomen. The pain was aggravated by inspiration but improved with Ibuprofen. It was not related to physical exertion nor feeding, and not associated with cough, fever, chills, night sweats, palpitations, nausea, vomiting, and shortness of breath. He also reported poor appetite, malaise, and generalized discomfort.

Of note, the patient had experienced a mechanical, ground-level near-fall with mild blunt impact to his anterior chest by a piece of furniture one week before his visit, which had resulted in a low-grade anterior chest wall pain that had resolved after treatment with topical analgesics. He had no other history of any chest or abdominal trauma or procedures in the past, and he denied any personal or family history of bleeding, clotting disorders, hepatobiliary or pancreatic disease, and malignancies. The patient was not on any prescription anticoagulants or multivitamins. He did not smoke nor use illicit drugs but consumed alcohol occasionally and denied any accidental exposure to any toxic chemicals in the recent past. His diet usually consisted of high-carbohydrate, high-calorie foods, and he rarely consumed fruits and vegetables.

Two months prior to his initial visit, the patient had upper experienced respiratory tract symptoms with an associated intermittent, nonproductive cough that had resolved completely without medication. Around the same time, he had also developed a relapse of his previous depressive symptoms, including poor sleep, lack of interest, attention, and poor appetite, which had made him skip meals. He denied any suicidal thoughts or ideation. The patient had consulted his primary care provider and received quetiapine for his symptoms.

**Figure 1:**
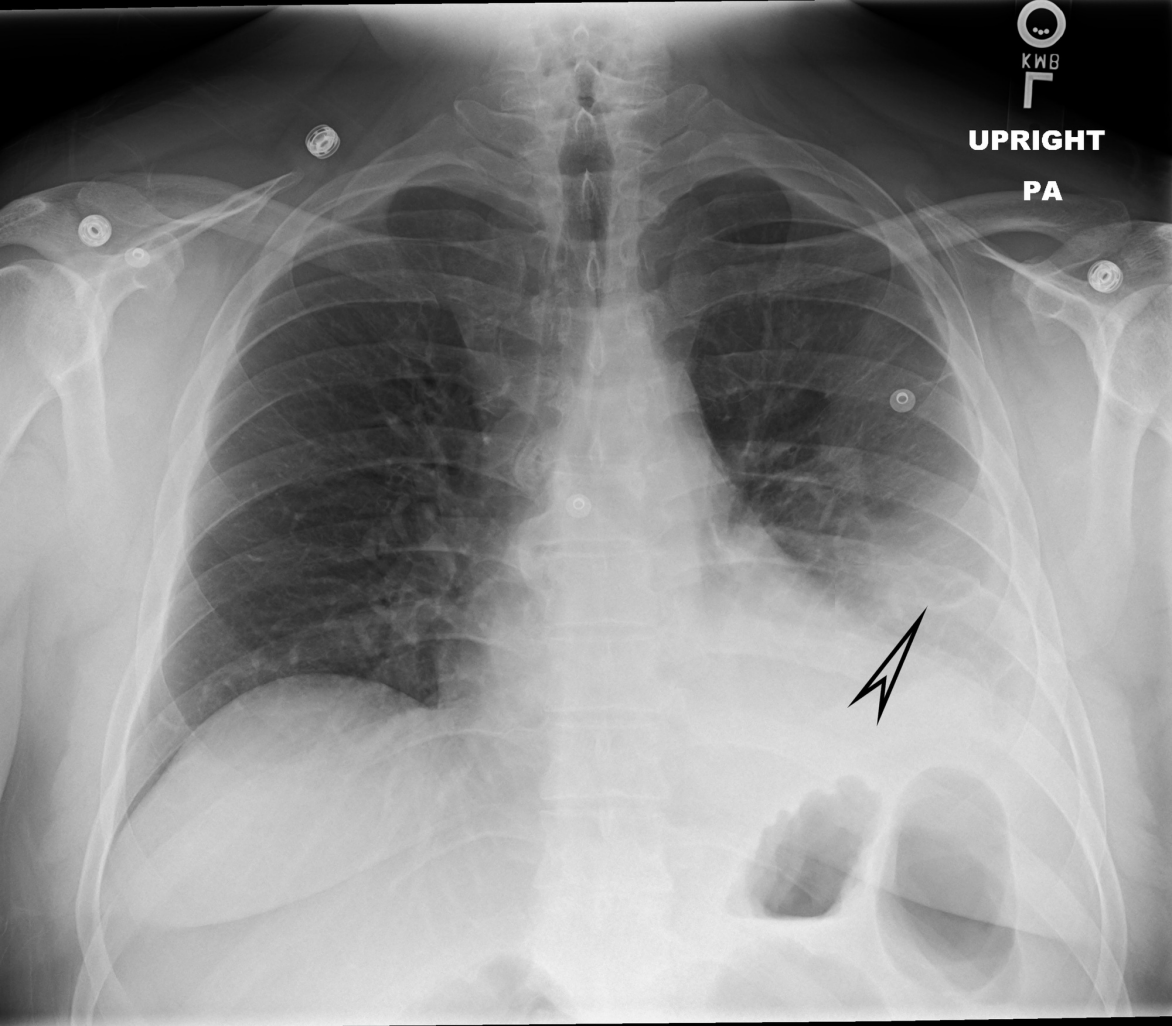
Chest X-ray highlighting a patchy infiltrate in the left lower lobe with a left pleural fusion and no pneumothorax (arrowhead)

**Figure 2:**
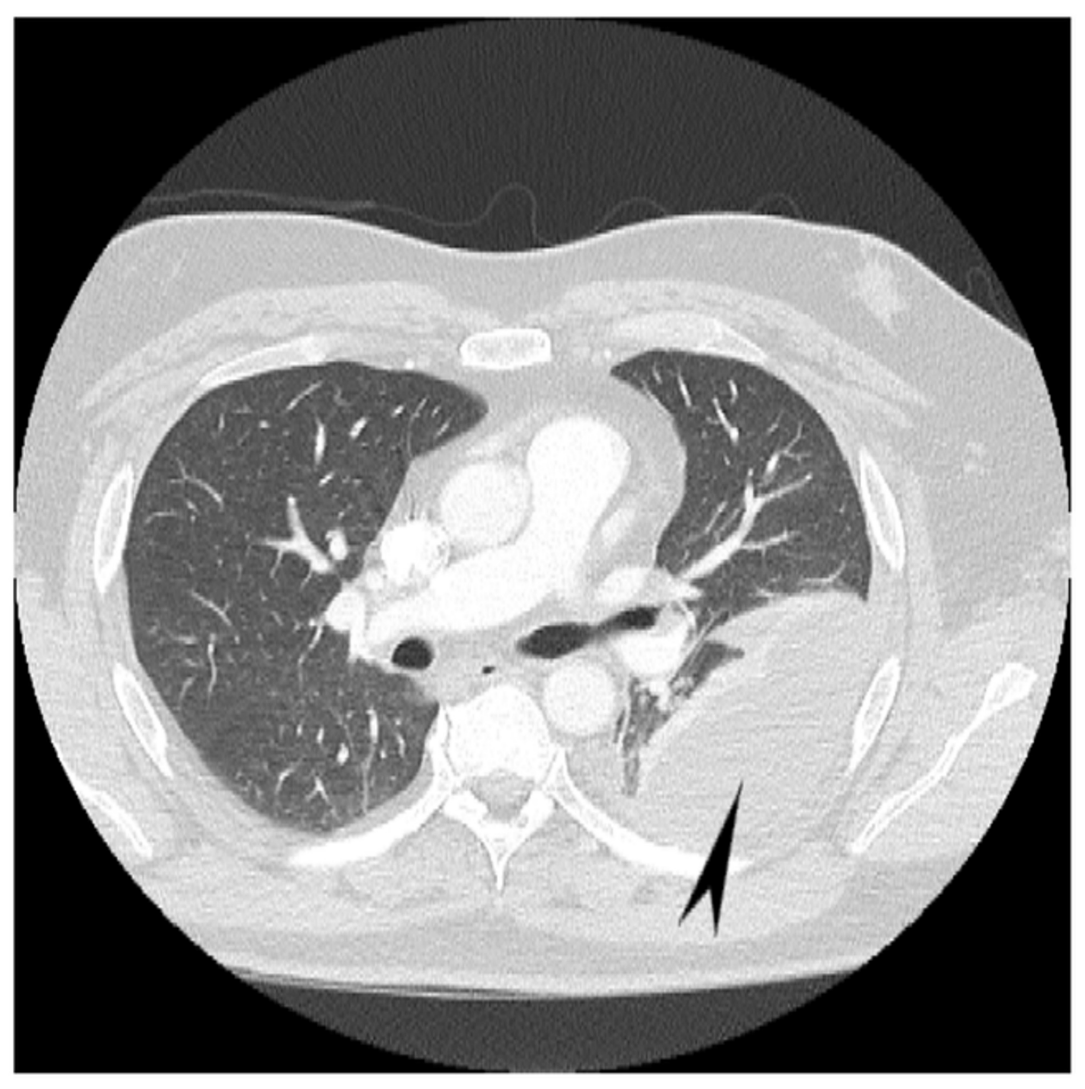
CT of the chest reveals a small-to-moderate loculated left pleural effusion with left basilar atelectasis (arrowhead) There is no CT evidence of pulmonary embolism CT: computed tomography

At the index visit, the patient’s vitals were normal, and his cardiac biomarkers were within normal limits. A preliminary electrocardiogram showed sinus tachycardia with nonspecific T wave abnormalities, and a corresponding chest X-ray revealed left-sided pleural effusion with patchy infiltrates (Figure 1). CT angiography scan of the chest was negative for pulmonary embolism but revealed small-to-moderate loculated left pleural effusion with left basilar atelectasis (Figure 2). These findings raised concerns for infectious, inflammatory, or malignant etiology. A diagnostic thoracentesis was performed, and 45 mL of hemorrhagic effusion was removed. The pleural fluid hematocrit was 3.3%, which was 10% of the serum hematocrit of 32.9% and supported a finding of hemorrhagic pleural effusion. The pleural fluid total red blood cell count was 359,816 cells/µL and total neutrophil count was 756 cells/µL. The pleural fluid was exudative with a pH of 7.3, elevated fluid lactate dehydrogenase (LDH)/serum LDH ratio of 3.3, and a significant fluid protein/plasma protein ratio of 0.67. All other pleural fluid studies including cytology were unremarkable. A pigtail catheter was placed and it drained approximately 1,500 mL of non-purulent hemorrhagic fluid over two days. The patient was initially started on empirical intravenous antibiotics that were later halted when aerobic and anaerobic cultures of the pleural fluid and blood came back negative for microbial growth.

Blood investigations showed normal white blood cell and platelet counts of 9.7 x 10^3/µL and 247 x 10^3/µL, respectively, normocytic anemia of 11.5 g/dL (normal range: 12.9-17.3), a normal level of pro-calcitonin of 0.06 ng/ml (normal range: 0.00-0.10), and elevated C-reactive protein of 23.6 mg/dl (normal range: 0-1.0), which was non-specific, and likely related to pleural hemorrhage-mediated inflammation. Immunological studies, including antinuclear antibodies and antiphospholipid antibody, were negative. Further serum analysis revealed normal albumin, fibrinogen, liver enzymes, and serum electrolyte levels, and his kidney function was within normal limits. Hematological analysis revealed an elevated international normalized ratio (INR) of 2.6 and an activated partial thromboplastin time (aPTT) of 48s (normal range: 23.5-32.1), which suggested a coagulopathy. A one-to-one mixing study produced a normalized aPTT of 32, which indicated the absence of clotting factor inhibitors. Factor II, VII, and X activities were all reduced at 61% (normal range: 70-160), 4% (normal range: 70-170), and 31% (normal range: 65-165), respectively, though factor V level was normal at 109% (normal range: 70-170). Micronutrient analysis of the serum revealed a low level of vitamin K1 level at <0.03 nmol/L (normal range: 0.10-2.20 nmol/L), indicative of an acquired vitamin K deficiency due to poor dietary intake in the setting of major depression. The patient was started on oral vitamin K supplementation and given nutritional counseling. After five days of vitamin K supplementation, the patient’s INR improved to 1.0, and his aPTT improved to 29.6, which confirmed vitamin K deficiency as the main cause of the coagulopathy. A follow-up chest CT showed considerable improvement in the amount of pleural effusion, and repeat labs revealed down-trending of his C-reactive protein from 23.6 to 1.7 mg/dl. The patient was discharged after nutritional counseling, and at the one-week follow-up, his coagulation studies remained within normal limits.

## Discussion

Vitamin K functions as a cofactor in the gamma-carboxylation of factors II, VII, IX, and X. When an individual becomes deficient in vitamin k, the decrease in post-translationally modified cofactors manifest as an initial increase in partial thromboplastin (PT) and with increased severity, aPTT. Very little dietary vitamin K is required for proper coagulation in humans, making it highly unusual for individuals to develop complications from vitamin K deficiency except in those with predisposing conditions [1,6-8].

Since primary vitamin K deficiency caused by an inherited enzyme deficiency is a profoundly rare condition reported in fewer than 30 people worldwide, inherited vitamin K deficiency is typically ignored in differential diagnoses [9]. While secondary vitamin K deficiency is common in neonates due to both exogenous and endogenous factors, it is rarely seen in healthy adults [1,6]. An adult requires about 100 micrograms of phylloquinone for maintenance of hemostasis, which is easily attained via a small portion of leafy greens, many animal products, and fermented foods [10]. There are very few documented cases of acquired vitamin K deficiency primarily due to poor dietary intake in otherwise healthy adults without predisposing conditions [8]. To the best of our knowledge, hemorrhagic effusion as a predominant symptom of acquired vitamin K deficiency in an adult has not been documented in the medical literature.

Initial coagulation studies of the patient revealed an elevated INR of 2.6 and an increased aPTT of 48. These symptoms suggested a range of possible diagnoses including inherited or acquired coagulopathies, coagulation factor deficiencies, or other conditions with hematologic abnormalities such as liver, hepatobiliary, or pancreatic disease, gastrointestinal malabsorption, disseminated intravascular coagulation, anticoagulant medications, or vitamin K deficiency, to name a few. By gathering a thorough history and reviewing the patient’s medical records, we noted that the patient had never been prescribed anticoagulant medications nor was he on any antibiotics, multivitamins, or supplements that could interfere with the coagulation pathway components. Hepatic disorders, biliary, or pancreatic disease were unlikely based on the patient’s initial symptoms, normal liver function, and pancreatic enzymes. The patient also did not fit the diagnosis for disseminated intravascular coagulation since he did not have any predisposing factors, his platelet counts and fibrinogen levels were within normal limits, and his coagulation studies normalized after the administration of oral vitamin K. Since the patient denied any diarrhea, constipation, or changes in bowel habits that might have suggested malabsorption syndrome, and since his immunological markers were negative, we ruled out any gastrointestinal or chronic inflammatory diseases. Mixing study results also did not suggest a coagulopathy due to coagulation factor inhibitors. His low serum level of factors II, VII, and X, as well as low serum vitamin K1 levels highly supported a diagnosis of vitamin K deficiency. These observations, coupled with the rapid normalization of the patient’s INR and aPTT over the course of five days with vitamin K supplementation, indicated that acquired vitamin K deficiency was the most likely etiology for the patient’s symptoms.

This case highlights that taking a thorough patient history along with reviewing the patient’s medical records remains an invaluable part of the clinical decision-making process. It also underlines the nutritional impact of depressive disorders, which should be explored during clinical encounters, as this may manifest as several macro- and micronutrient deficiencies. Along with severe depression, other conditions that would predispose a patient to poor nutritional intake should be considered in patients with unexplained coagulopathy.

## Conclusions

Hemorrhagic pleural effusion caused by vitamin K deficiency secondary to malnutrition in an adult is a rare presentation of this condition. Prompt identification of malnutrition-induced vitamin K deficiency, especially in the setting of major depression, obtained via a thorough patient history can assist in the timely management and complete reversal of the associated coagulopathy.

## References

[1] Pazirandeh S, Burns DL (2020). Pazirandeh S, Burns DL: overview of vitamin K. https://www.uptodate.com/contents/overview-of-vitamin-a?source=bookmarks.

[2] Sattler FR, Weitekamp MR, Ballard JO (1986). Potential for bleeding with the new beta-lactam antibiotics. Ann Intern Med.

[3] Hooper CA, Haney BB, Stone HH (1980). Gastrointestinal bleeding due to vitamin K deficiency in patients on parenteral cefamandole. Lancet.

[4] Shearer MJ, Bechtold H, Andrassy K (1988). Mechanism of cephalosporin-induced hypoprothrombinemia: relation to cephalosporin side chain, vitamin K metabolism, and vitamin K status. J Clin Pharmacol.

[5] Bettger WJ, Jones JP, Olson RE (1982). Effect of alpha-tocopherol and alpha-tocopherolquinone on vitamin K-dependent carboxylation in the rat. Fed Proc.

[6] Lippi G, Franchini M (2011). Vitamin K in neonates: facts and myths. Blood Transfus.

[7] Smith FR, Goodman DS (1976). Vitamin A transport in human vitamin A toxicity. N Engl J Med.

[8] Zekavat OR, Fathpour G, Haghpanah S, Dehghani SJ, Zekavat M, Shakibazad N (2017). Acquired vitamin K deficiency as unusual cause of bleeding tendency in adults: a case report of a nonhospitalized student presenting with severe menorrhagia. Case Rep Obstet Gynecol.

[9] Kanbur NO, Derman O, Kutluk T, Gürgey A (2004). Coagulation disorders as the cause of menorrhagia in adolescents. Int J Adolesc Med Health.

[10] Shearer MJ (2009). Vitamin K in parenteral nutrition. Gastroenterology.

